#  Mutation p.G83R in the transthyretin gene is associated with hereditary vitreous amyloidosis in Han Chinese families

**Published:** 2013-07-25

**Authors:** A-Mei Zhang, Hui Wang, Peng Sun, Qiu-Xiang Hu, Yuqing He, Yong-Gang Yao

**Affiliations:** 1 Key Laboratory of Animal Models and Human Disease Mechanisms of Chinese Academy of Sciences & Yunnan Province, Kunming Institute of Zoology, Kunming, Yunnan, China; 2 Kunming Aier Eye Hospital, Kunming, Yunnan, China; 3 University of Chinese Academy of Sciences, Beijing, China

## Abstract

**Purpose:**

Hereditary vitreous amyloidosis (HVA) is a genetic ophthalmological disorder. The purpose of this study was to investigate whether a mutation in the transthyretin (*TTR*) gene is associated with HVA in Han Chinese families.

**Methods:**

We performed clinical evaluation of three Han Chinese families with HVA and sequenced the entire exon of the *TTR* gene in probands and normal individuals from the families. The identified mutation was further genotyped in 196 unrelated healthy controls. Evolutionary conservation analysis and structural prediction were used to infer the potential pathogenicity of the mutation.

**Results:**

Clinical penetrance of HVA varied in the three families (11/30 in Family A, 8/83 in Family B, and 7/47 in Family C). A comprehensive medical examination of the patients showed no signs of abnormality except ophthalmologic symptoms, in which floccular turbidity and high echo in both vitreous bodies were observed in all probands. Further histochemical examination of the vitrectomy specimen with Congo red staining identified amyloid deposits. A heterozygous mutation c.307G>C (p.G83R) in exon 3 of the *TTR* gene was identified in all patients, but not in some unaffected family members. Screening of 196 unrelated normal controls revealed no presence of this mutation. This mutation changed the highly conserved glycine to arginine in the 83^rd^ position and altered the tertiary structure of the TTR protein.

**Conclusions:**

Mutation p.G83R in the TTR protein is associated with HVA in Chinese families. The seemingly specific distribution of this mutation in Han Chinese may be used for clinical diagnosis.

## Introduction

Familial amyloidotic polyneuropathy (FAP; MIM #105210) is an autosomal dominant neurodegenerative disease, which affects multiple tissues and organisms, such as the kidney, heart, and eyes. FAP occurs worldwide, and most patients develop clinical expression of the disorder in their 30s and 40s [1]. In some cases, vitreous opacity is the first and/or the merely clinical feature [2-4]. Several genes have been reported to be the pathogenic factors for this disorder. Among them, the transthyretin (*TTR*) gene is the most common; about 99% of cases are caused by mutations in this gene [5]. TTR is one of three plasma proteins with a tetrameric structure. The main function of TTR is to transport thyroid hormones and retinol (vitamin A) in vertebrata. TTR exists and is highly conserved only in vertebrates [6].

Since the first pathogenic mutation in the *TTR* gene was reported in 1984 [7], more than 100 mutations have been identified in patients with FAP . Mutation p.V30M is the most frequent and occurs in different populations, albeit it is mainly distributed in patients from Sweden, Japan, and Portugal [1,8]. Heterogeneity of clinical symptoms is common in families with FAP, even in the presence of the same TTR mutation [9]. The exact pathogenic mechanism of FAP caused by the TTR mutation(s) has not been well explained, but liver transplantation has been reported to relieve disease expression and prolong patients’ lifespans [10].

There have been limited reports of FAP in Chinese families [11-19]. In this study, we report three Chinese families with merely clinical expression of hereditary vitreous amyloidosis (HVA) and a *TTR* gene mutation, c.307G>C (p.G83R). Clinical examination and molecular analysis indicated that this rare mutation was the etiological factor for the three families and affected only the vitreous body.

## Methods

### Subjects

Nine patients with decreasing vision and six individuals without any ophthalmological disorders from three Han Chinese families were clinically evaluated, diagnosed, and recruited at the Yunnan Aier Eye Hospital. The general clinical information of these 15 subjects was listed in Table 1. The families were from Yunnan Province, China, and they have no self-reported affinity with each other. All patients developed blindness or had seriously visual impairment at the time when we collected the biological samples. Blood samples from nine patients and six normal individuals (each 3 ml) from the three families were collected by using vacuum blood collection tubes with K_2_EDTA, and stored at -20 °C prior to use. Written informed consent conforming to the tenets of the Declaration of Helsinki was obtained from each participant before the study was conducted. The institutional review board of the Kunming Institute of Zoology approved this study.

**Table 1:** Clinical phenotype for partial individuals of three Chinese families with vitreous amyloidosis.

Sample	Gender	Age	c.307G>C ^a^	Phenotype			
				Onset age	OS/OD^b^	OS/OD^c^	Operation
Family A							
II:7	Female	59	+	39	LP/ LP	0.1/ 0.15	Yes
III:14	Female	44	+	34	LP/ LP	0.15/ LP	Yes
III:15	Male	40	+	34	LP/ LP	0.15/ LP	Yes
III:17	Male	32	+	30	1.2/ LP	1.2/ 1.0	Yes
III:20	Male	34	+	33	1.0/ 0.4	1.0/ 0.4	Yes
Family B							
II:6	Female	76	-	-	-	-	No
III:21	Female	50	+	45	0.15/ LP	0.15/ 0.3	Yes
III:23	Male	47	-	-	-	-	No
III:25	Famle	44	+	40	LP/ 0.05	1.0/ 0.5	Yes
III:27	Female	38	+	-	-	-	No
IV:20	Female	26	+	-	-	-	No
IV:22	Male	25	-	-	-	-	No
Family C							
III:7	Male	59	+	40	LP/ LP	0.25/ 0.2	Yes
III:15	Male	43	+	40	LP/ 0.15	1.0/ 0.15	Yes
IV:1	Female	25	-	-	-	-	No

^a^mutation c.307G>C in the *TTR* gene is heterozygous in all patients. ^b^indicates the visual acuity before operation; OS and OD mean left and right eye, respectively. LP - light perception. ^c^indicates the visual acuity after operation; OS and OD mean left and right eye, respectively.

### Clinical examinations

Physical measurements for each family member were performed following the World Health Organization Multinational Monitoring of Trends and Determinants in Cardiovascular Disease Project standards. Ophthalmological measurements, including visual acuity test, intraocular pressure, visual field, color vision, slit-lamp photograph, dilated fundus examination, B-mode ultrasonography, electroretinogram, and visual evoked potential test, were analyzed blindly by two ophthalmologists. Routine blood tests and biochemical tests, urine routine examination, liver function test, kidney function tests, chest X-rays, electrocardiograph, and examinations of peripheral nerve system were performed to reveal potential dysfunction of these organs. Pathological tests of the vitreous bodies of the probands with Congo red staining were performed after vitrectomy and gas tamponade.

### Genetic analysis

Genomic DNAs were isolated from the whole blood of all examined individuals from the three families by using the AxyPrep Blood Genomic DNA Miniprep Kit (Axygen Biosciences, Union City, CA). We also isolated genomic DNA using the same approach from the whole blood of 196 unrelated healthy individuals from Yunnan Province who attended regular medical examinations. Three pairs of primers were designed to amplify and sequence four exons of the *TTR* gene in all examined family members (Table 2). The PCR amplification consisted of an initial denaturing cycle at 94 °C for 5 min, 30 cycles of denaturation at 94 °C for 30 s, annealing at 50 °C for 30 s, and extension at 72 °C for 30 s, followed by a final extension 72 °C for 5 min. We further sequenced the exon with the identified mutation in 196 healthy individuals by using the BigDye Terminator v3.1 Cycle Sequencing Kit (ABI, New York, NY) and the 3730 DNA Analyzer. Evolutionary conservation analysis of the TTR protein mutation was performed in ten vertebrate species (including *Homo sapiens, Pan troglodytes, Mus musculus, Rattus norvegicus, Bos taurus, Ovis aries, Sus scrofa, Gallus gallus, Xenopus laevis,* and *Danio rerio*). Swiss-PdbViewer software was used to predict whether the *TTR* mutation could change protein structure. In addition, we determined the mitochondrial DNA (mtDNA) haplogroup status of each maternally unrelated patient using the methods described in our previous study [20] to discern potential maternal kinship among the three families. In brief, each sample was amplified and analyzed for a 1.4 kb fragment (region 16024-850) that covers the entire mtDNA control region and was classified on the basis of haplogroup motif and (near-)matching with reported Chinese.

**Table 2:** Primers used for amplification and sequencing all four exons of the *TTR* gene.

Primers name	Primers sequences (5′-3′)	Amplifying region	Annealing temperature
Exon 1 & 2-F Exon 1 & 2-R	TGTTCCGATGCTCTAATCTCT TCTGCCTACGTTTTTCAATCT	Exon 1 & Exon 2^a^	50 °C
Exon 3-F Exon 3-R	CTACTTCTGACTTAGTTGAGG TGTATAATAGGAAAGGGAACC	Exon 3	50 °C
Exon 4-F Exon 4-R	TTCCTTCTGTTCAAACTGTTC CCTTGGAATGTGTCTTTTGCT	Exon 4	50 °C

^a^Exons 1 and 2 were amplified together by one pair of primers.

## Results

### Clinical features of three Chinese families with familial amyloidotic polyneuropathy

**Figure 1:**
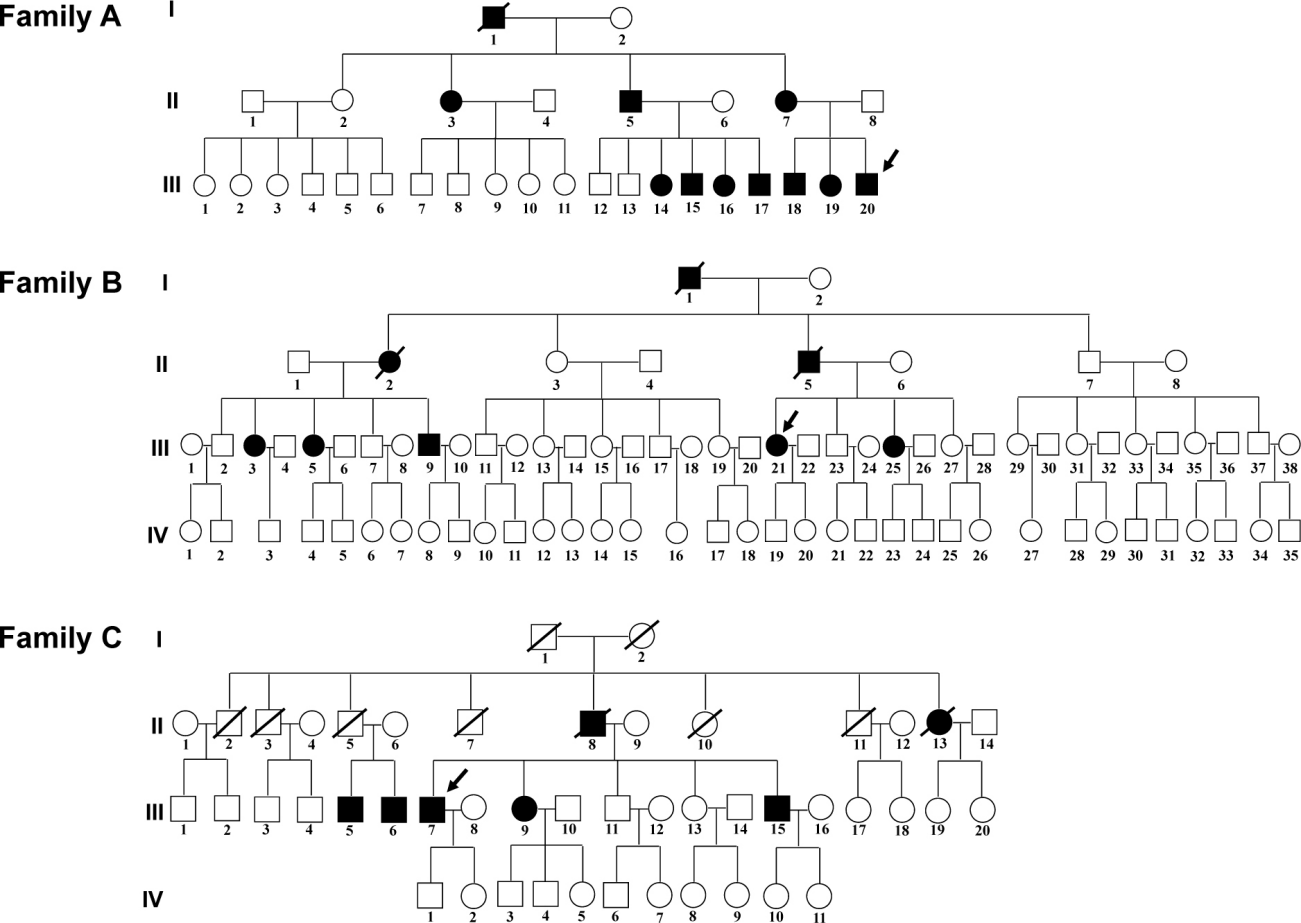
Pedigrees of three Chinese families with hereditary vitreous amyloidosis. The filled symbols indicate patients with vitreous amyloidosis. Penetrance of disease varied in Family A, Family B, and Family C. The probands are marked with arrows. The samples with a slash indicate deceased individuals.

In Family A, the proband (III:20) is a 34-year-old man who had had vision failure for 1 year. His visual acuity was gradually impaired: He saw floaters in both eyes, and finally developed complete vision loss. Eleven of 30 members in this family (including one deceased patient, I:1) experienced vision loss in their 30s (Figure 1A). The proband (III:21) of Family B is a 50-year-old woman who suffered vision problem in both eyes 5 years before and underwent an operation in her left eye 2 years before. Eight of 83 members of Family B had ophthalmologic features of FAP (Figure 1B). The proband (III:7) of Family C is a 59-year-old man who had vision loss at the age of 40. Seven of 43 individuals of Family C were confirmed to suffer visual problems.

**Figure 2:**
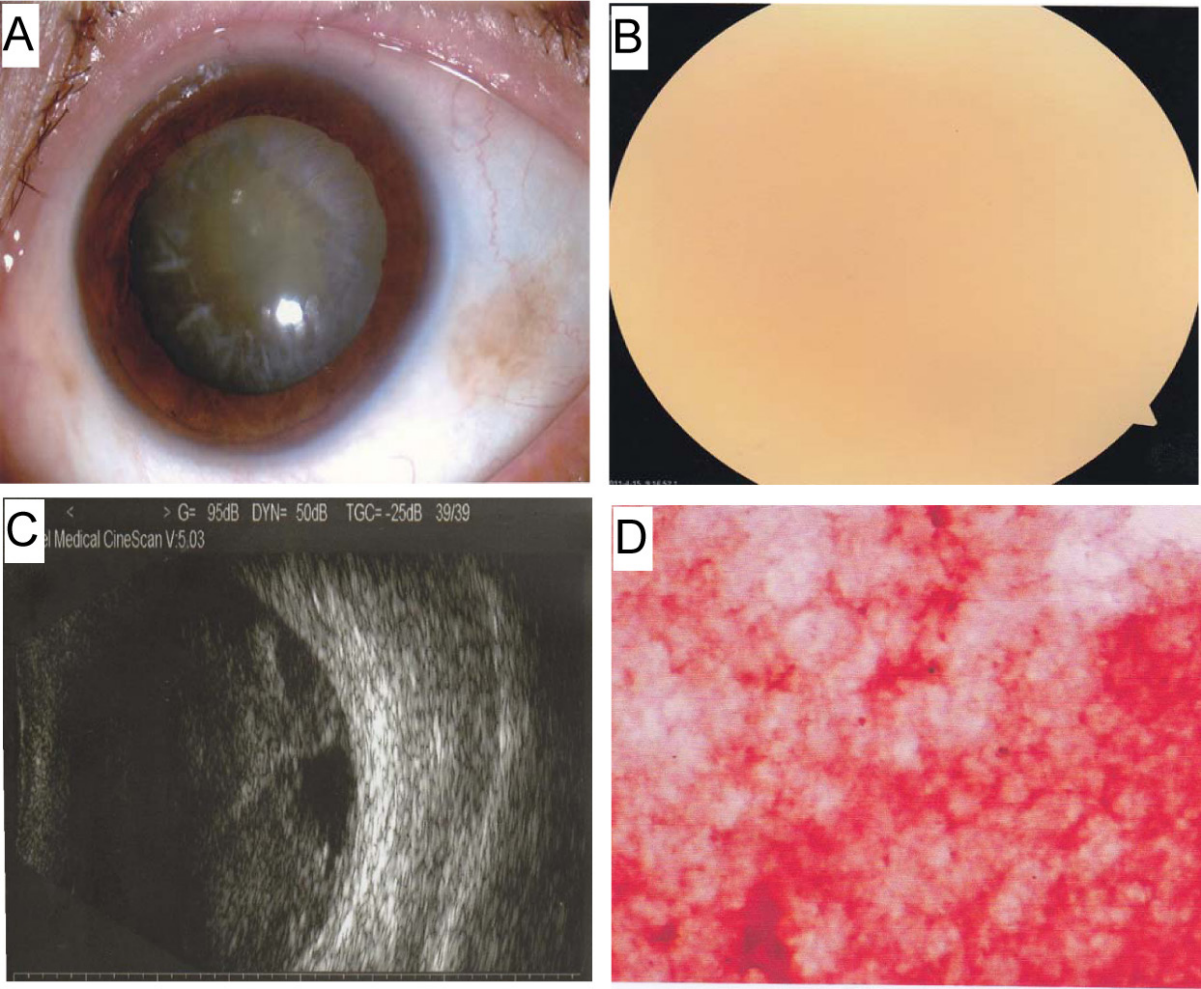
Ophthalmologic examinations of the left eye of the proband (III:20) from Family A. Slit-lamp photograph (**A**) and dilated fundus examination (**B**) show the floccular turbid of the vitreous body. B-mode ultrasonography shows the high echo in the vitreous body (**C**). Histochemical examination of vitrectomy specimen stained with Congo red shows amyloid deposits (in red; **D**).

The clinical phenotypes and examination results were similar in the three families. Though some patients in the three families, such as II:7 in Family A and the probands in Family B (III:21) and Family C (III:7), were older, they did not express any abnormality in other organs/tissues. The visual acuity of the patients ranged from light perception to 1.2 before surgery (Table 1), but the intraocular pressure was normal in all patients. Amyloidosis and high echoes were identified with the slit-lamp photograph test and B-mode ultrasonography (Figure 2), respectively. The results for the visual evoked potential test, retinal vessels, electroretinogram, and other routine measurements were normal in all patients. Eyesight of all patients was rescued after vitrectomy and gas tamponade. Pathological examination of Congo red staining showed amyloid deposition in the vitreous body of all probands (Figure 2). Other physical examinations, including routine blood tests and biochemical tests, urine routine examination, liver function test, kidney function tests, chest X-rays, electrocardiograph, and the examinations of peripheral nerve system revealed no potential dysfunction of these organs in the probands.

### Presence of the same transthyretin mutation p.G83R in three families

**Figure 3:**
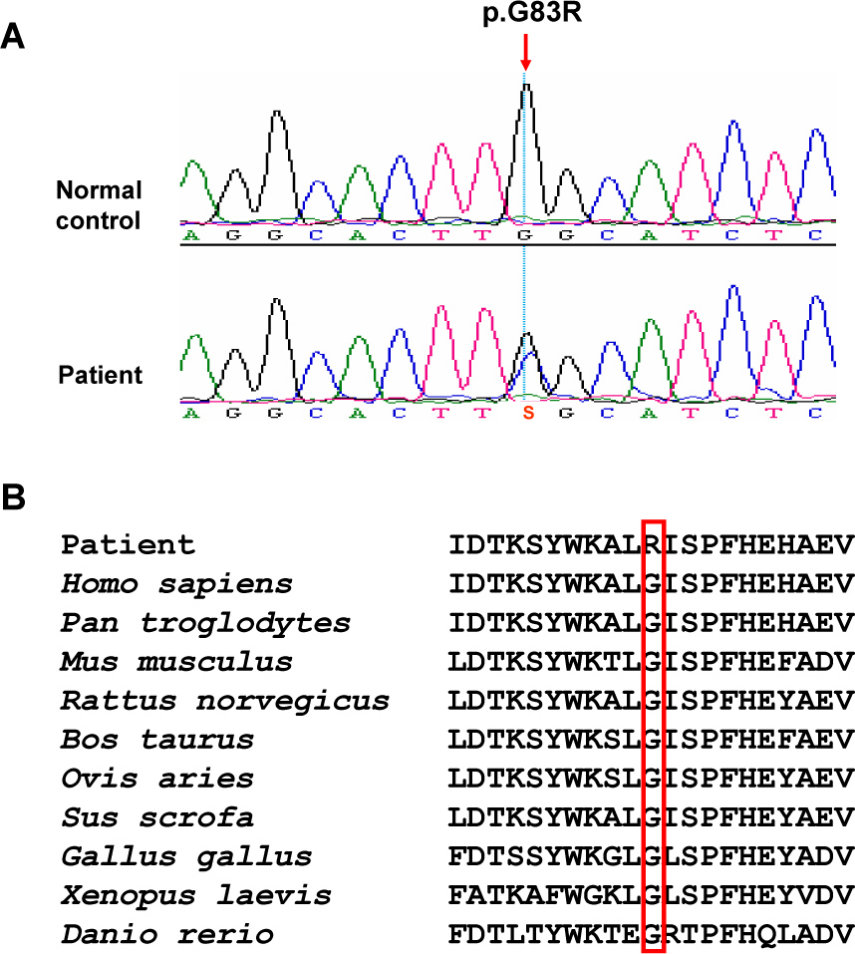
Analysis of the pathogenic mutation p.G83R of the transthyretin (TTR) protein in vertebrates. **A**: The sequencing electropherogram shows the mutated region in a normal control (III:23) and a patient (III:21) in Family B. **B**: Evolutionary analysis of position 83 in the TTR protein is shown. Protein sequences of *Homo sapiens* (CAG33189), *Pan troglodytes* (NP_001009137), *Mus musculus* (AAH24702), *Rattus norvegicus* (NP_036813), *Bos taurus* (NP_776392), *Ovis aries* (NP_001009800), *Sus scrofa* (NP_999377), *Gallus gallus* (NP_990666), *Xenopus laevis* (NP_001081348), and *Danio rerio* (AAI64894) were retrieved from GenBank.

**Figure 4:**
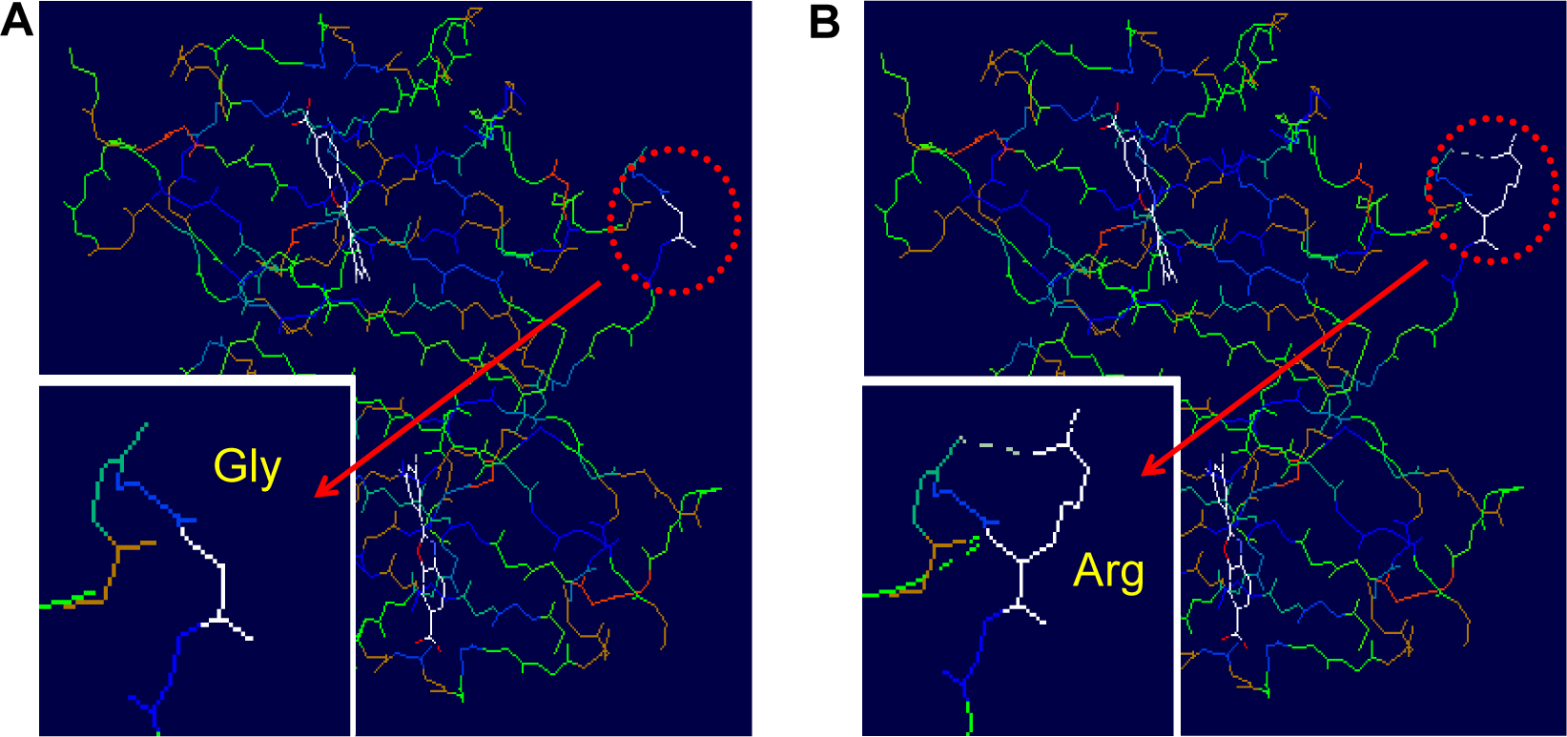
Comparison of homology modeling predicted transthyretin protein with and without mutant p.G83R. The side chain of the mutant was changed by p.G83R.

We amplified and sequenced the most common candidate gene, the *TTR* gene, and detected only a rare missense heterozygous mutation c.307G>C (p.G83R) in exon 3 of the probands of the three families (Figure 3A). After screening this mutation in all collected subjects from the three families and 196 unrelated healthy controls, we found that all patients, but not the normal controls (excluding individuals III:27 and IV:20 in Family B who had no ophthalmological or other symptoms but had mutation c.307G>C), harbored this mutation. Conservation analysis of ten vertebrate species indicated this rare mutation changed a highly conserved glycine to arginine in position 83 of the TTR protein (Figure 3B). As shown in Figure 4, the predicted three-dimensional structure of the TTR protein was changed by p.G83R.

mtDNA analysis of five maternally unrelated patients from the three families suggested that they belonged to different mtDNA haplogroups (Table 3): Patients III:14 and III:20 in Family A belonged to haplogroup D4b1 and B5a, respectively. Patient III:21 in Family B had a haplogroup status of G. Patients II:1 and III:1 in Family C belonged to haplogroups D4 and F, respectively. This result indicated that there is no potential maternal kinship among these families. The five mtDNA control region sequences have been deposited in GenBank under accession numbers JX988785-JX988789.

**Table 3:** The mtDNA control region sequence variations of some patients in the three families.

Family	Sample	Haplogroup	Sequence variants^a^
Family A	III:20	B5a	16,093, 16,140, 16183C, 16,189, 16,260, 16266G, 16,519, 73, 210, 249d, 263, 294, 309+C, 315+C, 709, 750
	III:14	D4b1	16,185, 16189d, 16,223, 16,319, 16,362, 73, 185, 189, 263, 315+C, 489, 523–524d, 750
Family B	III:21	G	16,185, 16,223, 16,311, 16,362, 16,526, 73, 263, 298, 315+C, 489, 523–524d, 709, 750
Family C	II:1	D4	16,051, 16,114, 16,223, 16,294, 16,311, 16,362, 73, 185, 263, 315+C, 489, 750
	III:1	F	16,086, 16,167, 16,203, 16,304, 16,318, 16,519, 73, 249d, 263, 315+C, 750

^a^mtDNA sequence variation was scored relative to the revise Cambridge Reference Sequence [29].

## Discussion

Though familial amyloidotic polyneuropathy occurs and is distributed worldwide, most cases have been reported in Japan, Portugal, and Sweden [21-23]. Some patients with FAP express complex clinical features, and the most frequently impaired tissue is the peripheral nerve system [24]. However, several patients experienced only decreasing eyesight and vitreous amyloidosis [2,25]. Here we reported three Chinese families with vitreous amyloidosis and the same rare heterozygous mutation p.G83R in the TTR protein. The affected members in Family A presented progressive visual disturbance in their 30s; the patients in Family B and Family C had visual failure in their 40s. All patients in the three families expressed only visual impairment, and their eyesight was rescued after ophthalmologic surgery.

Most of the cases of FAP are caused by a common mutation p.V30M of the TTR protein, and only a few have been caused by other rare mutations [8]. The mutant spectra of the TTR protein are diversiform in different populations [5]. In this study, the patients from the three Chinese families harbored the same mutation p.G83R. Intriguingly, this mutation was previously reported in three Chinese families with vitreous amyloidosis [16,26] (note that one of the two families reported in [26] was first reported in [27], but this was not mentioned in their later paper [26]; mutation p.G83R was named p.G103R in the family reported in [16]). Similar to our study, there are no other clinical features excluding decreasing vision and vitreous amyloidosis in the probands of these three reported Chinese families [16,26]. A search for literature identified three more Chinese families with HVA with different TTR mutations (p. K35Y and p.L55R were reported in [11]; p.R54G was reported in [14]). Evidently, mutant p.G83R is common in Chinese patients with HVA (six occurrences in nine families), similar to mutant p.V30M in patients with FAP. More Chinese patients with HVA, even sporadic cases of vitreous amyloidosis, should be screened to validate this pattern.

We determined the mtDNA background and haplogroups of five patients from the three families and excluded the possibility of potential maternal kinship among these families. This result suggests that mutation p.G83R might occur independently in the three families. The seemingly common distribution of this mutation in Han Chinese with HVA may be used for clinical diagnosis. Two individuals with c.307G>C (III:27 and IV:20) in Family B did not express any symptoms at the time of analysis. As these two individuals were younger than the average age of onset of the other patients in Family B, we speculate that the clinical onset of HVA is influenced by age, at least for members in this family. Detection of the mutation in these normal members may offer insight into early protection and prevention treatments. Conservative evolution analysis indicated that mutation p.G83R occurs in a highly conserved position and might impair the protein structure and function (Figure 4). We have speculated that the presence of mutation p.G83R might impair the tertiary structure of the TTR protein, leading to production of insoluble aggregates of the mutant protein [28]. Further experimental analyses should be performed to confirm the predicted pathogenicity of mutant p.G83R. Moreover, it is worth clarifying why the TTR mutant p.G83R influences only the vitreous body but not other tissues and how this mutation causes the disorder.

In short, we reported three Chinese families with HVA and mutation c.307G>C (p.G83R) in the *TTR* gene. Our study indicated that TTR mutant p.G83R might be a common pathogenic mutation in Chinese patients with HVA. Examining more families with HVA and functional characterization of mutant p.G83R is essential to further confirm our findings.
